# Quantitative Analysis of Anti-N and Anti-S Antibody Titers of SARS-CoV-2 Infection after the Third Dose of COVID-19 Vaccination

**DOI:** 10.3390/vaccines10071143

**Published:** 2022-07-18

**Authors:** Nuri Lee, Seri Jeong, Su Kyung Lee, Eun-Jung Cho, Jungwon Hyun, Min-Jeong Park, Wonkeun Song, Hyun Soo Kim

**Affiliations:** 1 Department of Laboratory Medicine, Hallym University Kangnam Sacred Heart Hospital, Hallym University College of Medicine, 1, Singil-ro, Yeongdeungpo-gu, Seoul 07441, Korea; nurilee822@hallym.or.kr (N.L.); hehebox73@hallym.or.kr (S.J.); mjpark@hallym.or.kr (M.-J.P.); swonkeun@hallym.or.kr (W.S.); 2 Department of Laboratory Medicine, Hallym University Dongtan Sacred Heart Hospital, Hallym University College of Medicine, 7 Keunjaebong-gil, Hwaseong-si 18450, Gyeonggi-do, Korea; sklee1217@naver.com (S.K.L.); ejlovi@hallym.or.kr (E.-J.C.); jungwonhyun@hallym.or.kr (J.H.)

**Keywords:** SARS-CoV-2, BNT162 vaccine, ChAdOx1 nCoV-19, COVID-19 booster shot

## Abstract

We quantitatively analyzed SARS-CoV-2 antibody levels in patients after two doses of the ChAdOx1 nCoV-19 vaccine and the third BNT162b2 booster. We obtained 255 serum samples from 149 healthcare workers 1 and 4 months after the third dose. Of the 149 participants, 58 (38.9%) experienced COVID-19 infection during the 4-month study period, with infection occurring 7–62 days before the second blood draw. Total antibody titers against the anti-spike (anti-S) and anti-nucleocapsid (anti-N) proteins of SARS-CoV-2 were measured using Elecsys Anti-SARS-CoV-2 S and Elecsys Anti-SARS-CoV-2 assays (Roche), respectively. The median anti-S antibody titer in the non-infected groups at 4 months after the third dose was significantly decreased compared to that at 1 month after the third dose (from 17,777 to 3673 U/mL, *p* < 0.001). The infected group showed higher median anti-S antibody titers at 4 months (19,539 U/mL) than the non-infected group (3673 U/mL). The median anti-N antibody titer in the infected group at 4 months after the third dose was a 5.07 cut-off index (79.3% positivity). Anti-N antibody titers in the infected group were correlated with the number of days after SARS-CoV-2 infection. These data provide useful information for determining quarantine strategies and fourth vaccination requirements.

## 1. Introduction

With the coronavirus disease 2019 (COVID-19) pandemic prolonged due to the frequent occurrences of severe acute respiratory syndrome coronavirus 2 (SARS-CoV-2) infections (even in vaccinated people), there has been growing interest in the quantitative value of SARS-CoV-2 antibodies present at the time of infection in vaccinated individuals. As of 6 May 2022, the third vaccination (booster) dose has been administered to approximately 64.61% of the total population in South Korea, and the cumulative number of SARS-CoV-2 confirmed cases is 17,464,782 [1].

As some countries are beginning to treat COVID-19 as an endemic disease, it is necessary to investigate the current status of SARS-CoV-2 antibody titers in infected and non-infected people vaccinated against COVID-19. Few studies have reported the antibody response in vaccinated individuals formerly infected with SARS-CoV-2. These studies showed that previously infected vaccinees have a significantly higher antibody response than vaccinated people who were previously uninfected [2,3,4,5,6]. However, these previous studies provide little information on the patients after administration of the third dose. Moreover, studies comparing the results between various target antigens of SARS-CoV-2 antibody measurement, such as anti-spike (anti-S) and anti-nucleocapsid (anti-N) proteins, are lacking.

For serological measurement of SARS-CoV-2 antibodies, tests that detect antibodies to S and N proteins, which are the most immunogenic proteins of SARS-CoV-2, are mainly used. Various studies reported the characteristics and quantitative values of the anti-S and anti-N antibodies against SARS-CoV-2 depending on whether the subjects were infected or vaccinated [7,8,9]. The spike (S) protein, which is present on the envelope of SARS-CoV-2, allows the virus to connect with human cells and has been reported to exert neutralizing effects in vitro. Anti-S may be useful as an indicator of an effective immune response, including vaccine effectiveness [7,10]. In contrast, nucleocapsid (N) proteins form a complex with genomic RNA and are involved in interactions with viral membrane proteins during virion assembly, increasing the efficiency of viral transcription and assembly [11,12]. Anti-S and anti-N have different characteristics depending on the time of SARS-CoV-2 infection, vaccination status, and severity of symptoms. Therefore, it is necessary to properly select the target protein according to the purpose of the test. In general, anti-S antibodies have been regarded as more important than anti-N antibodies when considering the efficacy of vaccines, as most COVID-19 vaccines target the S protein. However, anti-N is more influential as a biomarker of natural infection. Analysis of these two parameters is relatively simple, but the data produced appears to have a relatively high correlation with those obtained using conventional neutralizing tests, such as the plaque reduction neutralization test [13], and these factors have high utility in both the COVID-19 pandemic and endemic situations [14].

A few studies compared the patterns and quantitative values of anti-S and anti-N antibodies in SARS-CoV-2-infected and non-infected patients after the third vaccination dose. According to the Centers for Disease Control and Prevention, the third dose is recommended to further enhance or restore protection in most people aged 5 years and older; however, studies on the vaccine efficacy after the third dose are lacking. Therefore, we quantitatively analyzed SARS-CoV-2 antibodies according to the infection status after administration of two doses of the AstraZeneca ChAdOx1 nCoV-19 vaccine and a third dose of the Pfizer BNT162b2 vaccine, which was provided to most healthcare workers in this study.

## 2. Materials and Methods

### 2.1. Study Population and Sample Collection

A total of 149 healthcare workers from two university hospitals (Hallym University Dongtan Sacred Heart Hospital and Hallym University Kangnam Sacred Heart Hospital), who met the inclusion criteria, were enrolled in this study. The main inclusion criteria were as follows: age >20 years, eligibility for vaccination, and provision of informed consent, including acknowledgment of the purpose and design of this study. None of the participants had SARS-CoV-2 infection prior to enrollment in this study. Serum samples were collected from the participants to verify the SARS-CoV-2 antibody status at 1 month (T1) and 4 months (T2) after the third vaccination. The serum samples (≥300 μL volume) were aliquoted and stored at −70 °C until measurement. The third dose had been administered between 18 November and 23 December 2021. All enrolled participants had previously received two doses of the AstraZeneca ChAdOx1 nCoV-19 vaccine and a third dose of the Pfizer BNT162b2 vaccine. In total, 255 serum samples were collected from the 149 participants. Of these 149 participants, 91 had never been infected with SARS-CoV-2, whereas 58 had experienced SARS-CoV-2 infections. All patients with SARS-CoV-2 infection had been administered the third booster dose of the Pfizer BNT162b2 vaccine. This study was approved by the Institutional Review Boards of two hospitals, and informed consent was obtained from all participants in this study.

### 2.2. Measurement of Anti-SARS-CoV-2 Spike (Anti-S) and Anti-SARS-CoV-2 Nucleocapsid (Anti-N) Antibodies

The anti-S antibody concentration was measured in an Elecsys Anti-SARS-CoV-2 S assay on the Elecsys Cobas e801 platform (Roche Diagnostics, Mannheim, Germany). The anti-N antibody concentration was measured in an Elecsys Anti-SARS-CoV-2 assay on the same platform. These assays measure the total antibodies against the nucleocapsid protein and receptor-binding domain of the spike protein based on the electrochemiluminescence immunoassay (double-antigen sandwich principle). The cut-offs for anti-S and anti-N were 0.8 U/mL and 1.0 cut-off index (COI), respectively. A sample showing a value equal to or greater than the cut-off was interpreted as positive, and a sample showing a value below the cut-off was interpreted as negative. The positive rate was determined as the ratio of positive samples among all samples tested. A predefined master curve was adapted to the analyzer using relevant calibration materials. Controls for various concentration ranges were run individually at least once every 24 h. The values obtained were within defined limits.

### 2.3. Statistical Analysis

Chi-square tests were performed to compare nominal values, including sex and positivity of anti-N, between SARS-CoV-2-infected and non-infected participants. Mann–Whitney U tests were conducted to evaluate the statistical significance of differences in continuous values between SARS-CoV-2-infected and non-infected participants, including age, sampling days after the third vaccination and infection, and anti-S and anti-N titers. The anti-S and anti-N titers at T1 and T4 were also compared. Kruskal–Wallis tests were performed to analyze the effect of the classification factor on the period from the time of SARS-CoV-2 infection to blood collection for anti-N measurement. Spearman’s rank correlation coefficients were applied to assess correlations between the quantitative values of anti-S and anti-N antibodies as described previously [15]. MedCalc software version 19.8 (MedCalc Software Ltd., Ostend, Belgium) was used for these analyses.

## 3. Results

### 3.1. Characteristics of Participants

The characteristics and serological responses of the participants to SARS-CoV-2 infection are presented in Table 1. The median ages of the participants, with and without SARS-CoV-2 infection, were 39.5 and 32.0 years, respectively. The median sampling days after the third shot (T1 and T2) for all participants were 24.5 (range: 7.0–32.0) and 129.0 days (range: 103–151), respectively. No significant differences in age, sex, or sampling days between SARS-CoV-2-infected and non-infected participants were observed.

### 3.2. Qualitative and Quantitative Analyses of Anti-S Antibodies 1 and 4 Months after the Third Dose of COVID-19 Vaccination

**Figure 1:**
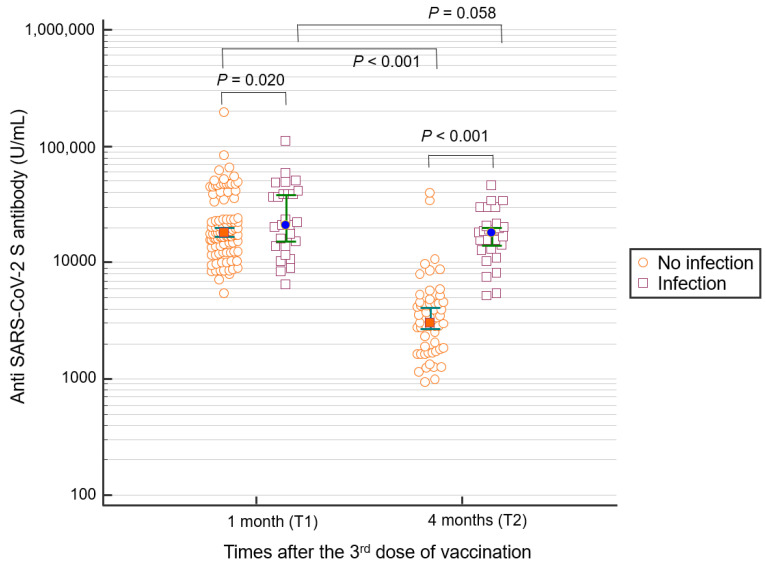
Anti-S antibody titer 1 (T1) and 4 months (T2) after the third vaccine dose. Participants with SARS-CoV-2 infection showed higher anti-S antibody levels than those without infection. In addition, in non-infected participants, the antibody level at 4 months after the third dose of vaccination was significantly lower than at 1 month.

The positive rate of anti-S antibodies after the third vaccine dose was 100% at T1 (1 month after the third dose) and T2 (4 months after the third dose). The median anti-S antibody titer both in the non-infected and infected groups at T2 was lower than that at T1 (Figure 1). For non-infected participants, the median anti-S antibody titer at T1 was 17,777 U/mL (range: 5458.0–193,360) and decreased to 3,673.0 (927–39,740) U/mL at T2 (*p* < 0.001). In infected participants, the median anti-S antibody titers at T1 and T2 were 23,215 (6455–110,600) and 19,538.5 (1138–111,580) U/mL, respectively (*p* = 0.058). The median anti-S antibody titer for SARS-CoV-2–infected participants at T2 was statistically significantly higher than that for non-infected participants (non-infected: 3676, infected: 19,538.5 U/mL, *p* < 0.001).

### 3.3. Qualitative and Quantitative Analyses of Anti-N Antibodies 4 Months after Third Dose of COVID-19 Vaccination

**Figure 2:**
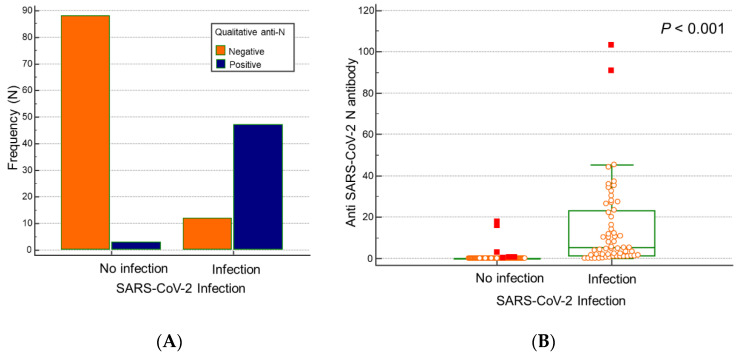
Quantitative and qualitative serological responses for anti-N antibodies after 4 months after the third dose of vaccination (T2). (**A**) Number of participants with anti-N positivity was significantly higher in the SARS-CoV-2-infected group than in the non-infected group. (**B**) The SARS-CoV-2-infected group showed a higher anti-N antibody titer than the non-infected group.

Among the participants with SARS-CoV-2 infection, 46 participants were positive in the anti-N antibody assay; in the non-infected group, three participants were positive in the anti-N assay (Figure 2A). The positivity rates at 4 months after the third dose of vaccination (T2) for SARS-CoV-2 infected and non-infected participants were 79.3% (46/58) and 3.3% (3/91), respectively. The median titer of anti-N antibodies at 4 months after the third dose (T2) was 5.07 COI (range: 0.06–103.0) in the infected group (Figure 2B), which was significantly higher than that in the non-infected group (0.07 COI, range: 0.06–17.7, *p* < 0.001).

### 3.4. Quantitative Analysis of Anti-N and Anti-S SARS-CoV-2 Antibodies Based on Duration of SARS-CoV-2 Infection

**Figure 3:**
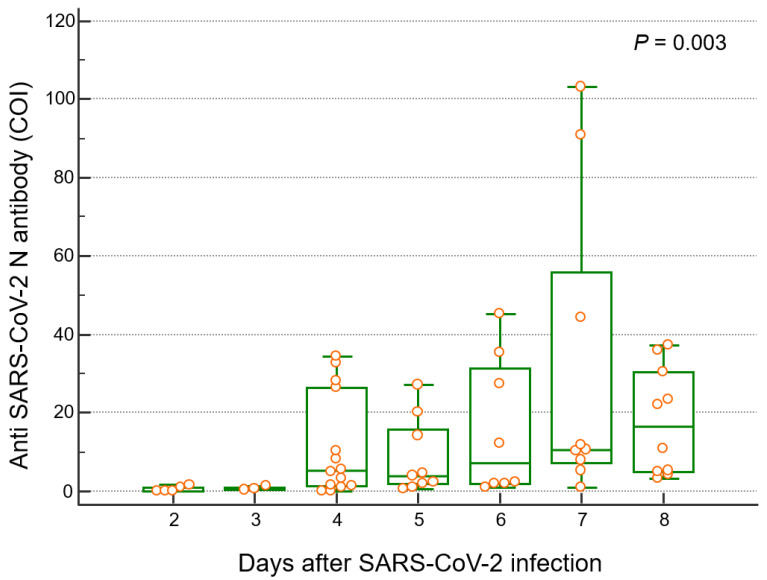
Distribution of anti-SARS-CoV-2 N antibody levels according to the number of days after SARS-CoV-2 infection. Anti-N antibody at different time points classified by five days is described.

The anti-N antibody levels based on the number of days after SARS-CoV-2 infection are presented in Table 2 and Figure 3. The median duration from the COVID-19 diagnosis to T2 blood sampling in the infected group was 22.5 days (interquartile range (IQR): 17.0–33.0, range: 7.0–62.0). After SARS-CoV-2 infection, the anti-N antibody levels were significantly correlated with the period of infection (r^2^ = 0.3331, *p* = 0.0099). The participants were divided into groups based on the period from SARS-CoV-2 infection to blood collection; the group from 6 to 10 days was designated as D1, and groups D2–D6 had a 5-day interval between each, and the group after 36 days was designated as D7. Among these participant groups, the anti-N titer showed the highest median value (16.5 COI, IQR: 7.2–55.9, Table 2) in D7. The median anti-N antibody levels for participants in D1, D2, D3, D4, D5, D6, and D7 were 0.11, 0.64, 5.01, 3.88, 7.25, 10.5, and 16.5 COI, respectively (Table 2).

### 3.5. Correlation between Anti-N and Anti-S Antibody Levels

**Figure 4:**
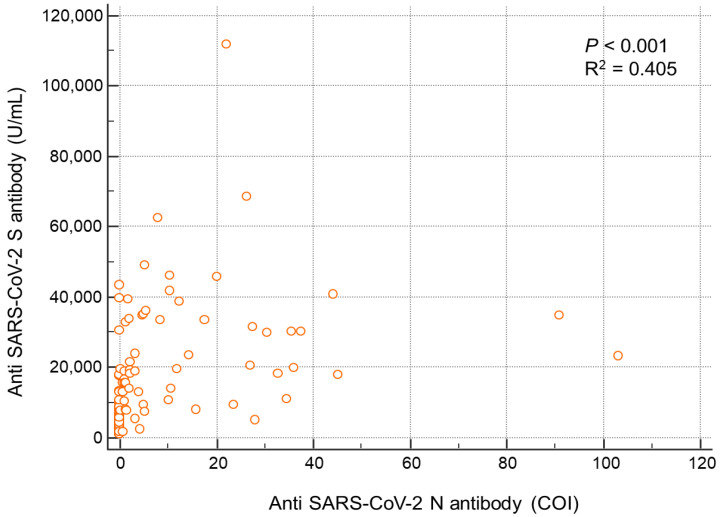
Correlation between anti-S and anti-N antibody levels in each participant.

The correlation between the quantitative values of anti-S and anti-N antibodies in the infected group is shown in Figure 4. Anti-S was positively correlated with anti-N (r^2^ = 0.405, *p* < 0.001) (Figure 4).

## 4. Discussion

In this study, after the third dose of SARS-CoV-2 vaccination, the median anti-S antibody titer at 4 months was decreased compared to the titer at 1 month in the non-infected group. In addition, the anti-S antibody titer after 4 months was significantly higher in the infected group than in the non-infected group. Anti-N antibodies were increased in the infected group, and the value increased over the 36 days after infection.

As the fully vaccinated population has increased [1], it is important to accurately assess their immunity. Antibody testing for SARS-CoV-2 has been reported to be useful for evaluating the baseline seroprevalence of SARS-CoV-2 and identifying low- or non-responders to COVID-19 vaccines [14]. Particularly, as the COVID-19 pandemic continues, an antibody test can be helpful as an auxiliary test for differential diagnosis of SARS-CoV-2 and for determining whether additional vaccination is required [16].

According to a previous study of antibody titers after the third vaccination, the anti-S antibody titers at 20, 30, 60, and 90 days peaked at 30 days [17]. Moreover, at 6 months after the third dose of the BNT162b2 vaccine, the level of anti-S antibodies in individuals with a negative SARS-CoV-2 infection history was reported as 379–2960 AU/mL [18]. Humoral immunity, represented by the neutralization capacity via the ACE2 receptor competition, has also been reported to decrease starting at 6 months after the booster vaccination [19]. In this study, similar to previous studies, a comparison of anti-S antibodies at 1 and 4 months after the third vaccination revealed that anti-S antibodies had decreased at 4 months compared to 1 month. Participants were infected an average of 106.0 days (range: 77–136 days) after the third vaccination, and the decrease in the anti-S antibody titer was not significant in infected participants compared to that in non-infected participants. In addition, 39.4% of patients were infected with SARS-CoV-2 when they were expected to have a relatively high titer after the third vaccination, and the antibody titers in these patients were not lower than those in non-infected participants. A third vaccination has been suggested to reduce the rates of both confirmed and severe COVID-19 [20]. However, for the Omicron variant, it has been reported that even two BNT162b2 vaccinations may not adequately protect against severe disease [21,22]. The results of this study also showed that the antibody level before infection did not strongly protect against the Omicron variant.

Anti-N antibodies have been reported as indicators of natural infection [2,8]. Azak et al. reported that the positive rates of anti-N in vaccinated and infected patients were 51.2% and 98.8%, respectively [8]. In another study, anti-N showed significantly higher antibody titers in previously infected individuals compared to vaccinees without a history of infection [23]. Nevertheless, the duration of immunity or the neutralizing effect of anti-N is unknown. Moreover, compared to studies of anti-S antibodies, there are fewer studies involving long-term assessment of anti-N titers after vaccination and/or SARS-CoV-2 infection. Similar to anti-S, anti-N antibody levels are considered to peak at 6–7 weeks after the first or second vaccination or after infection [24]. In this study, the anti-N antibody titer was significantly higher in infected patients than in non-infected participants at 4 months after the third vaccination, and the positivity rates were 3.2% in non-infected participants and 79.3% in infected participants. Participants who showed discrepancies between infection and the anti-N test results were further analyzed. For infected participants who were negative for anti-N antibodies, the average duration of infection was 3.6 days, suggesting that the titer had not increased sufficiently to be detected. Additionally, four patients had an anti-N antibody titer of ≥0.9, which was close to the cut-off value. In contrast, three non-infected participants were positive for anti-N antibodies; further investigation of their family history and symptoms suggested that they may have experienced asymptomatic COVID-19 infections.

In this study, anti-N antibodies positively correlated with the infection days and positively correlated with anti-S in the infected group. To date, few studies have compared anti-S and anti-N antibodies in SARS-CoV-2-infected patients after the third vaccine dose. Moreover, the clinical application of anti-S and anti-N antibodies in SARS-CoV-2-infected patients has not been established. We found that anti-N antibodies are not affected by vaccines and can better reflect the patient’s immunity after SARS-CoV-2 infection. Currently, infections in vaccinated people are increasing, and measurements of anti-N together with anti-S are advantageous for accurately determining the patient’s current immunity. Anti-N antibodies are expected to be useful for long-term assessment in clinical practice, particularly for infected patients. In addition, simultaneous measurement of anti-S and anti-N in clinical practice can help determine whether a fourth vaccination dose should be administered.

A limitation of this study involved the measurement of anti-S and anti-N antibodies when antibody formation had not reached a peak, and the antibody titer may have been higher than the reported value if the subjects were reassessed after some time. The positivity rate of anti-N antibodies and the average value of antibody titers measured in this study may have been underestimated. In addition, it was difficult to compare the differences in results obtained using other assays because we used assay kits from only one manufacturer. This study was targeted at health workers; the proportion of females was high, and individuals over 60 years of age were excluded. Caution is required in interpreting the sex distribution, and further studies of the elderly and children are needed.

## 5. Conclusions

In conclusion, we investigated SARS-CoV-2 antibody titers at 4 months after the third COVID-19 vaccination dose. Anti-S and anti-N antibody titers in infected participants were compared with those in non-infected participants. These data provide useful information for the long-term assessment of patients with COVID-19 and the identification of patients who require additional vaccination.

## Figures and Tables

**Table 1:** Characteristics and demographics of participants with the third dose of COVID-19 vaccination, regarding SARS-CoV-2 infection.

	SARS-CoV-2 Infection (−) (*n* = 91)	SARS-CoV-2 Infection (+) (*n* = 58)	*p*-Value
Age	32.0 (23.0–59.0)	39.5 (22.0–60.0)	0.8658
Sex (male: female)	85:6	51:7	0.2498
Sampling days after the third vaccination (T1) *	25.0 (7–32)	23.5 (14–32)	0.8318
Sampling days after the third vaccination (T2) *	129 (103–134)	129 (113–151)	0.8725
Post-infection days after the third vaccination	N/A	106 (77–136)	N/A
T2 sampling days after infection	N/A	22.5 (7–62)	N/A

Values are presented as the median (range). * Serum samples were collected from the participants 1 month (T1) and 4 months (T2) after the third vaccination. Abbreviation: N/A, not applicable.

**Table 2:** Quantitative values of anti-N COI of participant groups divided based on the period from SARS-CoV-2 infection to blood collection.

Groups * (Days after Infection)	*n*	Minimum (COI)	Maximum (COI)	Median (IQR) (COI)	*p* < 0.05 from Groups
6–10 (D1)	5	0.07	1.59	0.11 (0.08–1.13)	D3, D4, D5, D6, D7
11–15 (D2)	3	0.19	1.22	0.64 (0.30–1.08)	D3, D5, D6, D7
16–20 (D3)	14	0.06	34.5	5.27 (1.05–22.4)	D1, D2, D6
21–25 (D4)	9	0.83	27.2	3.88 (1.88–15.8)	D1
26–30 (D5)	8	0.91	45.2	7.25 (2.03–31.6)	D1, D2
31–35 (D6)	9	0.94	103.0	10.5 (7.20–55.9)	D1, D2, D3
36–62 (D7)	10	3.30.	37.4	16.5 (4.92–30.5)	D1, D2

* Groups were assigned based on the period from the time of SARS-CoV-2 infection to blood collection for anti-N antibody measurements. Abbreviations: COI, cutoff index; IQR, interquartile range.

## Data Availability

Lee N., Kim H.S. 2022. Dataset of anti-N and anti-S antibodies against SARS-CoV-2 after the third dose of COVID-19 vaccination. Deposited in https://dataverse.harvard.edu/dataset.xhtml?persistentId=doi%3A10.7910%2FDVN%2FJAZ67R&version=DRAFT (accessed on 31 May 2022).
